# Blood plasma B vitamins in depression and the therapeutic response to electroconvulsive therapy

**DOI:** 10.1016/j.bbih.2020.100063

**Published:** 2020-03-28

**Authors:** Karen M. Ryan, Kelly A. Allers, Andrew Harkin, Declan M. McLoughlin

**Affiliations:** a Trinity College Institute of Neuroscience, Trinity College Dublin, Dublin 2, Ireland; b Department of Psychiatry, Trinity College Dublin, St. Patrick’s University Hospital, James Street, Dublin 8, Ireland; c Central Nervous System Disease Research, Boehringer Ingelheim Pharma GmbH + Co. KG, Birkendorferstrabe 65, Biberach a.d. Riss, Germany; d Neuropsychopharmacology Research Group, School of Pharmacy and Pharmaceutical Sciences & Trinity College Institute of Neuroscience, Trinity College, Dublin 2, Ireland

**Keywords:** Vitamin B, Depression, Electroconvulsive therapy, IL-6, CRP, Kynurenine pathway, Pyridoxal 5′-phosphate, Nicotinamide

## Abstract

A growing body of research has indicated a role for B vitamins in depression, with some previous studies suggesting that B vitamin status in patients with depression can impact on antidepressant response. Here we aimed to investigate B vitamin plasma concentrations in medicated patients with depression (*n* ​= ​94) compared to age- and sex-matched healthy controls (*n* ​= ​57), and in patients with depression after electroconvulsive therapy (ECT) in a real-world clinical setting. Our results show that nicotinamide (vitamin B3), N1-methylnicotinamide (vitamin B3 metabolite), and pyridoxal 5′-phosphate (PLP; vitamin B6) concentrations were significantly reduced in patients with depression compared to controls. The Cohen’s d effect sizes for nicotinamide, N1-methylnicotinamide, and PLP were moderate–large (−0.47, −0.51, and −0.59, respectively), and likely to be of clinical relevance. Functional biomarkers of vitamin B6 status (PAr index, 3-hydroxykynurenine: hydroxyanthranilic acid ratio, 3-hydroxykynurenine: xanthurenic acid ratio, and HKr) were elevated in depressed patients compared to controls, suggestive of reduced vitamin B6 function. Over 30% of the patient cohort were found to have low to deficient PLP concentrations, and exploratory analyses revealed that these patients had higher IL-6 and CRP concentrations compared to patients with PLP levels within the normal range. Treatment with ECT did not alter B vitamin concentrations, and B vitamin concentrations were not associated with depression severity or the therapeutic response to ECT. Overall, reduced plasma PLP, nicotinamide, and N1-methylnicotinamide concentrations could have wide ranging effects on pathways and systems implicated in depression. Further studies are required to understand the reasons why patients with depression present with low plasma B vitamin concentrations.

## Highlights

• Plasma vitamins B3 and B6 are reduced in depressed patients versus controls.
• Reduced B3 and B6 is likely to be clinically relevant (moderate-large Cohen’s d).
• 32% of depressed patients had low/deficient pyridoxal 5′-phosphate (PLP; B6) levels.
• Low PLP in depressed patients was associated with high IL-6 and CRP levels.
• Treatment with a course of ECT had no effect on plasma B vitamin concentrations.

## 1 Introduction

The role of nutrition in mental health is currently coming to the fore in psychiatric research, with a recent meta-review of meta-analyses indicating that nutrient supplements may represent suitable adjunctive treatments for depression (Firth et al., 2019). A growing body of research has indicated a role for B vitamins in depression. In this regard, several studies have suggested that low dietary intake of B vitamins, in particular folate (vitamin B9) and vitamin B12, is associated with depression (Bender et al., 2017; Herbison et al., 2012; Murakami and Sasaki, 2010) while, in contrast, high dietary intake of B vitamins may be protective against depressive symptoms (Moore et al., 2019; Skarupski et al., 2010). Moreover, polymorphisms in genes involved in B vitamin absorption (e.g., *methylene tetrahydrofolate reductase*; *MTHFR*) have been linked to an increased incidence of psychiatric and cognitive disorders (Mitchell et al., 2014).

The vitamin B complex consists of a group of eight structurally dissimilar water-soluble vitamins that perform essential and closely related roles in cellular functioning. Namely, these are thiamine (vitamin B1), riboflavin (vitamin B2), niacin (vitamin B3), pantothenic acid (vitamin B5), pyridoxal 5′-phosphate (PLP; vitamin B6), biotin (vitamin B7), folate (vitamin B9), and vitamin B12 (Kennedy, 2016). Flavin mononucleotide represents the active form of riboflavin (vitamin B3), while nicotinamide, the amide form of niacin, represents the active form of vitamin B3, which can be further metabolized to N1-methylnicotinamide by N-methyltransferase. PLP represents the active form of vitamin B6, which is transported around the body in the form of pyridoxal (PL) and is catabolized to pyridoxic acid (PA) for excretion. These vitamins are essential for physiological function but are primarily not synthesized endogenously and are instead acquired from the diet in small quantities. B vitamins are typically synthesized by plants but can also be consumed indirectly from plants via foods of animal origin, such as meat, dairy, and eggs, with some metabolism by the intestinal microbiota (Kennedy, 2016; Yoshii et al., 2019). Several B vitamins are involved in mitochondrial function and one-carbon metabolism, resulting in the formation of amino acids, fatty acids, and pyrimidines. They are also involved in both the folate and methionine cycle as well as glucose metabolism, all of which are essential to cellular function. Dysfunction of these mechanisms has been linked to depression (see Ueland et al., 2017 and Mikkelsen et al., 2016 for review). Deficiency in any one B vitamin can also contribute to the accumulation of homocysteine, which can have negative cellular consequences and has been postulated to play a role in the development of depression (Folstein et al., 2007).

Each B vitamin is actively transported across the blood-brain barrier or choroid plexus, illustrating the importance of their role in brain function (Kennedy, 2016). In the brain, B vitamins have many roles, including energy production, DNA/RNA synthesis and repair, epigenetic regulation, and neurotransmitter synthesis, with adequate levels of all B vitamins required for proper physiological and neurological functioning (Kennedy, 2016; Mikkelsen et al., 2016).

**Fig. 1:**
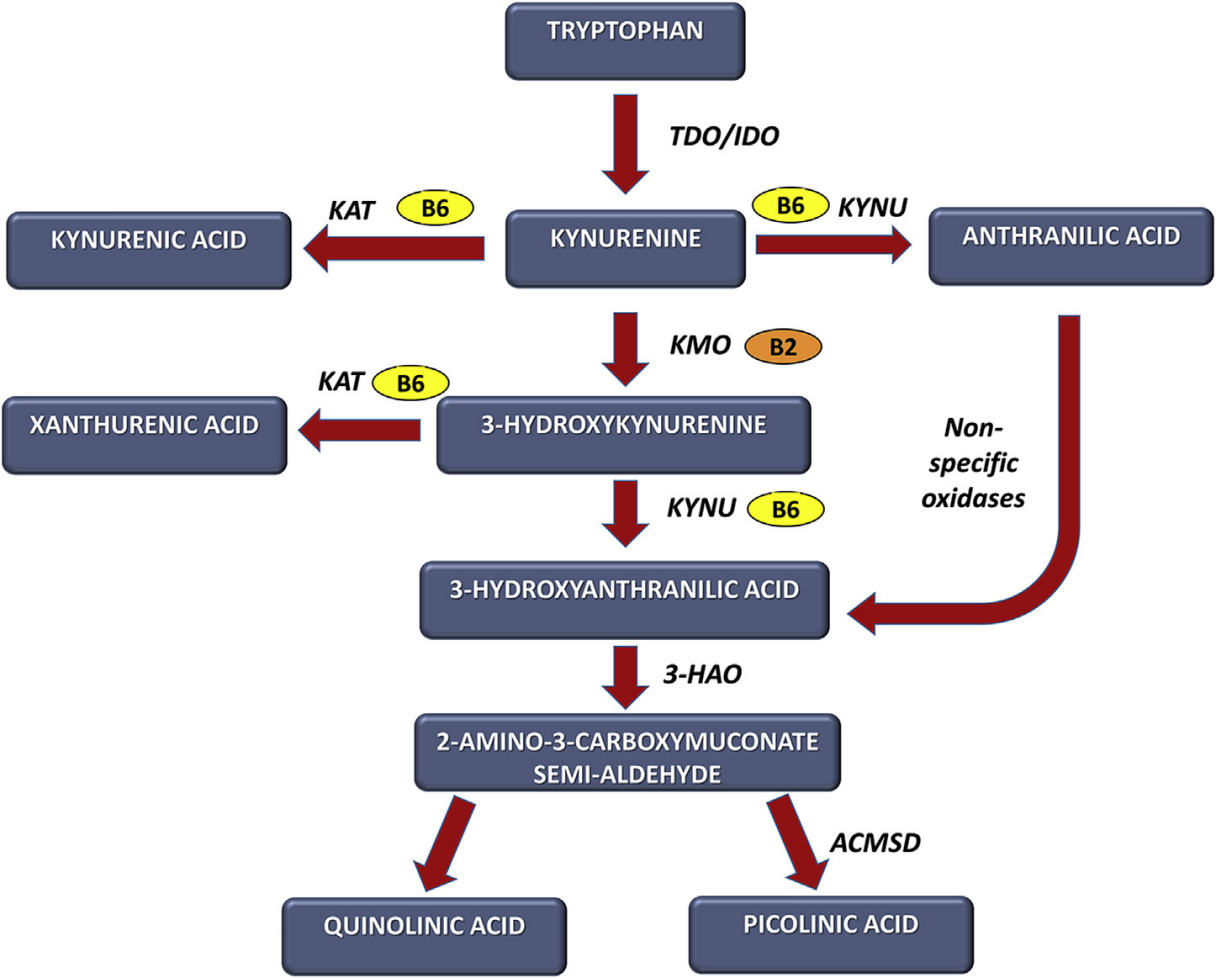
Role of B vitamins in the kynurenine pathway vitamin B2 acts as a co-factor for kynurenine 3-monooxygenase (KMO) and is implicated in the conversion of kynurenine to 3-hydroxykynurenine. Vitamin B6 (PLP) acts as a co-factor for the enzyme kynurenine aminotransferase (KAT), which converts kynurenine to kynurenic acid and 3-hydroxykynurenine to xanthurenic acid. Vitamin B6 (PLP) also acts as a co-factor for kynureninase (KYNU), which converts kynurenine to anthranilic acid and hydroxykynurenine to hydroxyanthranilic acid. (Adapted from Ryan et al. (2020)). The ratios of hydroxykynurenine (HK) to xanthurenic acid (XA) or 3-hydroxyanthranilic acid (HAA), i.e., HK:XA or HK:HAA, reflect the activity of the B6-dependent enzymes KAT and KYNU, respectively (Ueland et al., 2015). The HK ratio, which is calculated as the ratio of the kynurenine pathway metabolites hydroxykynurenine to the sum of kynurenic acid (KYNA) ​+ ​xanthurenic acid ​+ ​3-hydroxyanthranilic acid ​+ ​anthranilic acid (AA), i.e. HK:(KYNA ​+ ​XA ​+ ​HAA ​+ ​AA), reflects intracellular vitamin B6 status, with an increased ratio indicative of decreased PLP activity (Ulvik et al., 2020). Abbreviations: AA, anthranilic acid; ACMSD, aminocarboxymuconate semialdehyde decarboxylase; HAA, 3-hydroxyanthranilic acid; 3-HAO, 3-hydroxyanthranilic acid 3,4-dioxygenase; HK, hydroxykynurenine; IDO, indolamine 2,3-dioxygenase; KAT, kynurenine aminotransferase; KMO, kynurenine 3-monooxygenase; KYNA, kynurenic acid; KYNU, kynureninase; PLP, pyridoxal 5′-phosphate; TDO, tryptophan 2,3-dioxygenase; XA, xanthurenic acid.

B vitamins participate in a wide range of neurochemical pathways implicated in depression and other psychiatric conditions, such as the serotonergic, noradrenergic, dopaminergic, glutamatergic, GABAergic, and cholinergic systems (Ducker and Rabinowitz, 2017; Mikkelsen et al., 2016). They also play an important role in the tryptophan-kynurenine pathway. For example, vitamin B2 acts as a co-factor for kynurenine 3-monooxygenase (KMO), while vitamin B6, in the form of PLP, acts as a co-factor for enzymes such as kynurenine aminotransferase (KAT) and kynureninase (KYNU), which convert kynurenine to kynurenic acid (KYNA) or anthranilic acid (AA), respectively, and hydroxykynurenine (HK) to xanthurenic acid (XA) or hydroxyanthranilic acid (HAA), respectively (Fig. 1; Majewski et al., 2016; Ueland et al., 2017). Notably, we and others have recently shown concentrations of tryptophan and kynurenine pathway metabolites, including XA, HAA, KYNA, and AA, to be reduced in patients with depression (Aarsland et al., 2019; Arnone et al., 2018; Ogyu et al., 2018; Ryan et al., 2020). Many studies have also shown links between inflammation and depression (Chamberlain et al., 2019; Enache et al., 2019; Hiles et al., 2012; Howren et al., 2009; Lamers et al., 2019; Mikkelsen et al., 2017; Valkanova et al., 2013), which may be linked to vitamin B status since both human and animal studies have shown that B vitamin concentrations are related to immune cell activity and inflammatory mediator concentrations, e.g., C-reactive protein (CRP), interleukin-6 (IL-6) (see Mikkelsen et al., 2017; Ueland et al., 2017).

Previous studies have shown that vitamin B status in patients with depression can impact on antidepressant response. Vitamin B9 (folate) represents the most widely assessed vitamin supplement for mental disorders (Firth et al., 2019). For example, when used as an adjunctive treatment to selective serotonin reuptake inhibitors/serotonin norepinephrine reuptake inhibitors (SSRIs/SNRIs) B9 is associated with a greater reduction in depressive symptoms (Ginsberg et al., 2011). A study of patients with low-normal vitamin B12 levels also found that B12 supplementation enhanced the response to treatment with SSRIs (Syed et al., 2013), while a similar effect has additionally been found for adjuvant daily thiamine, which, compared to placebo, has been reported to improve the effects of SSRIs within the first six weeks of initiation (Ghaleiha et al., 2016). Moreover, a systematic review by Almeida et al. (2015) suggested that the long-term consumption of vitamins B12 and folate may decrease the risk for depression relapse and the onset of clinical depression in people at high risk, though supplementation was not shown to be effective in the short-term. A previous study also showed that baseline serum vitamin B12 levels are significantly higher in medicated patients with bipolar depression who respond to a single infusion with ketamine (Permoda-Osip et al., 2013).

Electroconvulsive therapy remains the most acutely effective antidepressant treatment for severe, often treatment-resistant depression (UK ECT Review Group, 2003). However, to our knowledge, no study has yet assessed vitamin B complex concentrations in severely depressed patients referred for a course of ECT or the impact of vitamin B status on the therapeutic response to ECT. We therefore hypothesized that plasma vitamin B concentrations would be lower in patients with depression compared to controls, and that patients who remitted following a course of ECT would have higher vitamin B concentrations than those who did not. We also explored the relationship between vitamin B concentrations and depression severity, inflammatory mediators, and tryptophan-kynurenine metabolites.

## 2 Methods

### 2.1 Participants

Ethical approval for this study was granted by St Patrick’s University Hospital Research Ethics Committee, and the study was performed in accordance with the Declaration of Helsinki (World Medical Association, 2013). All participants provided written informed consent.

All patients with depression were recruited in St Patrick’s Mental Health Services as part of the EFFECT-Dep Trial (Enhancing the Effectiveness of ECT in Severe Depression; NCT01907217), a pragmatic, patient- and rater-blinded, non-inferiority trial comparing the effects of brief-pulse twice-weekly moderate-dose bitemporal (1.5 ​× ​seizure threshold) and high-dose unilateral (6 ​× ​seizure threshold) ECT in real-world practice (Semkovska et al., 2016). Patients were maintained on pharmacotherapy-as-usual throughout the ECT course. Inclusion criteria were: >18 years old; referred for ECT for a major depressive episode diagnosed using the Structured Clinical Interview for DSM-IV Axis I Disorders (First et al., 1996); and pre-treatment Hamilton Depression Rating Scale 24-item version (HAM-D24) (Beckham and Leber, 1985) score ≥21. Exclusion criteria were: substance misuse in the previous six months; medically unfit for general anesthesia; dementia or other axis I diagnosis; and ECT in the previous six months. ECT was administered twice-weekly with hand-held electrodes, as previously described (Semkovska et al., 2016). Methohexital (0.75 mg/kg–1.0 ​mg/kg) and succinylcholine (0.5 mg/kg–1.0 ​mg/kg) were used, respectively, for anesthesia and muscle relaxation.

Healthy controls were recruited through local newspaper and social media advertisements. Participants with an immune disorder (e.g., systemic lupus erythematosus, psoriasis) or major neurological illness (e.g., Parkinson’s disease, stroke) were excluded from the present analysis, as previously reported (Ryan et al., 2020).

Clinical and demographic data were documented. Patients were also asked about any family history of depression (i.e., first or second degree relative). Depression severity and response to ECT were assessed using the HAM-D24. Remission was defined as ≥60% reduction in HAM-D24 score and a score ≤10 for two weeks.

### 2.2 Blood sampling and laboratory analyses

Fasting blood samples were taken 07:30–09:30 before the first ECT treatment, and at 1–3 days after the final ECT session. Fasting control blood samples were taken 07:30–09:30 on assessment days. Peripheral blood (10 ​mL) was collected into K_2_EDTA tubes (BD, UK). Tubes were centrifuged at 2000 ​rpm for 10 ​min ​at room temperature, following manufacturer’s guidelines, and plasma was stored in aliquots at −80 ​°C until analysis.

Plasma samples (100 ​μl) were analyzed at BEVITAL AS (Bergen, Norway; www.bevital.no). LC/MS was used to quantify circulating concentrations of thiamine (vitamin B1), thiamine monophosphate (vitamin B1 metabolite), riboflavin (vitamin B2), flavin mononucleotide (vitamin B2 metabolite), nicotinamide (vitamin B3), N1-methylnicotinamide (vitamin B3 metabolite), pyridoxal 5′-phosphate (PLP; vitamin B6), pyridoxal (PL; vitamin B6), and 4-pyridoxic acid (PA; vitamin B6 catabolite). The % coefficients of variation (% CV) were as follows: thiamine ​= ​6.2, thiamine monophosphate ​= ​10.6, riboflavin ​= ​5.7, flavin mononucleotide ​= ​15.8, nicotinamide ​= ​10.2, N1-methylnicotinamide ​= ​5.1, PLP ​= ​5.2, PL ​= ​5.9, PA ​= ​8.1. The lower limits of quantification were as follows: thiamine ​= ​0.25 ​nmol/L, thiamine monophosphate ​= ​0.5 ​nmol/L, riboflavin ​= ​0.2 ​nmol/L, flavin mononucleotide ​= ​0.4 ​nmol/L, nicotinamide ​= ​20 ​nmol/L, N1-methylnicotinamide ​= ​5 ​nmol/L, PLP ​= ​0.2 ​nmol/L, PL ​= ​0.2 ​nmol/L, PA ​= ​0.5 ​nmol/L.

Tryptophan and kynurenine pathway metabolites were also assessed at BEVITAL AS and IL-6, IL-10, tumor necrosis factor alpha (TNF-α), and CRP protein concentrations were analyzed using the MSD® MULTI-SPOT Assay System (Meso Scale Diagnostics LLC, USA) as previously described (Ryan et al., 2020).

To determine B vitamin function, we assessed the PAr index, the HK ratio, and the HK:XA and HK:HAA ratios (see Fig. 1 for reference). The PAr is calculated as the ratio of the vitamin B6 catabolite PA to the sum of its active form PLP and its transport form PL, i.e., PA:(PL ​+ ​PLP). An increase in the PAr is indicative of altered vitamin B6 homeostasis towards increased B6 catabolism (Ueland et al., 2015). The ratios of the kynurenine pathway metabolites HK to XA and HAA were also assessed, i.e., HK:XA and HK:HAA. Increased HK:XA and HK:HAA ratios are indicative of increased HK in the plasma owing to a reduction in the activity of the B6-dependent enzymes KAT and KYNU, respectively (Ueland et al., 2015). The HK ratio, which is calculated as the ratio of the kynurenine pathway metabolite HK to the sum of KYNA, XA, HAA, and AA, i.e. HK:(KYNA ​+ ​XA ​+ ​HAA ​+ ​AA), has recently been demonstrated to represent a sensitive and specific indicator of intracellular vitamin B6 status, with an increased ratio indicative of decreased PLP activity (Ulvik et al., 2020).

### 2.3 Statistical analysis

Data were analyzed using SPSS version 26 (IBM Corporation, NY, USA). Clinical and demographic data were examined using independent t-tests or Chi-square (Χ^2^) tests. Data were tested for normality using the Kolmogorov-Smirnov test and Q-Q plots. Data were subsequently log_10_ transformed for analysis where appropriate. A general linear model, both unadjusted and adjusted for potential confounders, was used to determine differences between groups. Age, sex, body mass index (BMI), and smoking status were included as potential confounders since these have been linked to changes in vitamin B status (Vermaak et al., 1990). Educational attainment differed significantly between control and depressed groups and was thus included as a potential confounder. We also adjusted for diabetes, cardiovascular disease, and the use of non-steroidal anti-inflammatory drugs (NSAIDs) owing to their links to vitamin B deficiencies (Chang et al., 2013; Dziegielewska-Gesiak et al., 2019; Friedman et al., 2004; Kennedy, 2016; Liu et al., 2017; Ma et al., 2017; Merigliano et al., 2018; Shen et al., 2010; Ulvik et al., 2016; van Oijen et al., 2004). For pre-/post-ECT analyses, we included polarity of depression, presence of psychosis, and baseline depression severity, where appropriate. Data not normally distributed after log_10_ transformation were analyzed using non-parametric methods (Mann-Whitney U, Kruskal Wallis, Wilcoxon Signed Rank test). Pearson’s r or Spearman’s ρ correlation tests were used to assess relationships between continuous variables. Data are presented as mean (standard deviation, SD) or number (%) per group where appropriate. To account for multiple comparisons, a Benjamini-Hochberg correction (Hochberg and Benjamini, 1990), which provides better type I error control than more conservative approaches, was applied using a false discovery rate (FDR) of 0.05.

## 3 Results

### 3.1 Participants

For this study, plasma samples were available from 113/138 patients with depression recruited to the EFFECT-Dep Trial. We excluded any participants (controls and patients) with an immune disorder (*n* ​= ​14) or major neurological illness (*n* ​= ​8) from analyses. The groups were then balanced for age and sex. A total of 94 patients with depression and 57 healthy controls were included in the final statistical analyses. The demographic and clinical characteristics are summarized in Table 1.

**Table 1:** Demographic and clinical characteristics of participants.

	Depressed Baseline/Pre-ECT (n ​= ​94)	Controls (n ​= ​57)	Statistical test
Age, years	55.48 (14.72)	51.74 (11.47)	t ​= ​1.74, *p* ​= ​0.08
Sex, No. (%)			
Male	36 (38.30)	20 (35.09)	χ^2^ ​= ​0.16, *p* ​= ​0.69
Female	58 (61.70)	37 (64.91)	
BMI	26.76 (5.01)	25.54 (4.08)	t ​= ​1.55, *p* ​= ​0.12
Smokers, No. (%)	40 (42.55)	7 (12.28)	χ^2^ ​= ​15.17, *p* ​< ​0.001
Education, No. (%)			
Primary	15 (15.96)	4 (7.02)	χ^2^ ​= ​35.27, *p* ​< ​0.001
Secondary	53 (56.38)	9 (15.79)	
Tertiary & Quaternary	26 (27.66)	44 (77.19)	
Bipolar depression, No. (%)	21 (22.34)		
Psychotic depression, No. (%)	21 (22.34)		
Baseline HAM-D24	31.01 (6.57)	2.82 (1.99)	t ​= ​38.75, *p* ​< ​0.001
Post-ECT HAM-D24	7.64 (7.55)		
Electrode placement, No. (%)			
Unilateral	48 (51.06)		
Bitemporal	46 (48.94)		
Number of ECT sessions	7.97 (2.46)		
Responders, No. (%)	60 (63.83)		
Remitters, No. (%)	50 (53.19)		
Psychotropic medications, No. (%) taking			
SSRI	22 (23.40)		
SNRI	48 (51.06)		
TCA	25 (26.60)		
MAOI	10 (10.64)		
Mirtazapine	32 (34.04)		
Bupropion	1 (1.06)		
Lithium	34 (36.17)		
Sodium Valproate/Lamotrigine	6 (6.38)		
Antipsychotics	65 (69.15)		
Benzodiazepines	49 (52.13)		
Non-benzodiazepine hypnotics	56 (59.57)		
Pregabalin	5 (5.32)		
Other	16 (17.02)		

Data are presented as means with standard deviations (SD) or number (%) per group where appropriate.

Abbreviations: BMI, body mass index; ECT, electroconvulsive therapy; HAM-D24, Hamilton depression rating scale, 24-item version; MAOI, monoamine oxidase inhibitor; SNRI, serotonin-norepinephrine reuptake inhibitor; SSRI, selective serotonin reuptake inhibitor; TCA, tricyclic antidepressant.

### 3.2 Patients with depression compared to healthy controls

#### 3.2.1 B vitamin concentrations in patients with depression compared to controls

Table 2 shows the plasma B vitamin concentrations from patients with depression compared to healthy controls. Unadjusted analyses show that flavin mononucleotide (B2), nicotinamide (B3), N1-methylnicotinamide (B3), and PLP (B6) concentrations were lower in depressed patients compared to controls, while PA (B6) and PL (B6) concentrations were increased in depressed patients. However, after adjustment for the potential confounders specified above and for multiple comparisons, only nicotinamide, N1-methylnicotinamide, and PLP remained significantly different between depressed patients and controls. The Cohen’s d effect sizes for nicotinamide, N1-methylnicotinamide, and PLP were moderate–large (−0.47, −0.51, and −0.59, respectively), and likely to be of clinical relevance.

**Table 2:** B vitamin plasma concentrations in healthy controls and medicated patients with depression.

	Controls (*n* ​= ​57)	Depressed Baseline (*n* ​= ​94)	Cohen’s d	Unadjusted Statistics	Adjusted Statistics^a^
*B vitamins*					
Thiamine (B1)	4.25 (2.00)	5.80 (8.13)	0.26	*U* ​= ​2520, *p* ​= ​0.542; *p*_*FDR*_ ​= ​0.542	
Thiamine Monophosphate (B1)	7.50 (2.24)	6.99 (2.80)	−0.20	*F*_1,149_ ​= ​2.85, *p* ​= ​0.094; *p*_*FDR*_ ​= ​0.12	*F*_1,132_ ​= ​1.84, *p* ​= ​0.18; *p*_*FDR*_ ​= ​0.18
Riboflavin (B2)	18.63 (16.55)	17.36 (17.87)	−0.07	*U* ​= ​2901, *p* ​= ​0.394; *p*_*FDR*_ ​= ​0.44	
Flavin Mononucleotide (B2)	13.27 (12.77)	9.99 (10.61)	−0.28	*U* ​= ​3588.5, *p* ​< ​0.001; ***p***_***FDR***_***=* 0.002**	
Nicotinamide (B3)	1126.58 (314.31)	968.59 (355.20)	−0.47	*F*_1,149_ ​= ​7.64, *p* ​= ​0.006; ***p***_***FDR***_***=* 0.01**	*F*_1,132_ ​= ​4.74, *p* ​= ​0.031; ***p***_***FDR***_***=* 0.04**
N1-methylnicotinamide (B3)	149.92 (73.41)	115.06 (63.62)	−0.51	*F*_1,149_ ​= ​10.51, *p* ​= ​0.001; ***p***_***FDR***_***=* 0.002**	*F*_1,132_ ​= ​5.73, *p* ​= ​0.018; ***p***_***FDR***_***=* 0.036**
Pyridoxal 5ʹ-phosphate (B6)	79.90 (46.56)	51.24 (50.71)	−0.59	*F*_1,149_ ​= ​35.47, *p* ​= ​1.79 ​× ​10^−8^; ***p***_***FDR***_***=* 0.002 × 10**^**−7**^	*F*_1,132_ ​= ​13.70, *p* ​= ​0.0003; ***p***_***FDR***_***=* 0.001**
Pyridoxic Acid (B6)	32.70 (15.63)	37.09 (100.83)	0.06	*U* ​= ​3272, *p* ​= ​0.023; ***p***_***FDR***_***=* 0.03**	
Pyridoxal (B6)	16.68 (11.15)	18.40 (76.96)	0.03	*U* ​= ​4325.5, *p* ​< ​0.001; ***p***_***FDR***_***=* 0.002**	

*Ratios indicative of B vitamin function*					
PAr	0.37 (0.27)	0.54 (0.27)	0.63	*U* ​= ​1503, *p* ​< ​0.001	
HK:XA	2.69 (1.46)	5.17 (3.23)	0.99	*U* ​= ​984, *p* ​= ​7.7 ​× ​10^−11^	
HK:HAA	1.13 (0.32)	1.45 (0.72)	0.57	*U* ​= ​1994, *p* ​= ​0.009	
HKr	0.34 (0.08)	0.46 (0.18)	0.86	*U* ​= ​1409, *p* ​= ​0.000001	

Data are presented as mean (SD) nmol/L.

*p*_FDR_ represents the adjusted p-value following Benjamini-Hochberg analysis (only B vitamins were included in this analysis and not functional ratios). *p*_FDR_ highlighted in bold attains statistical significance.

PAr = PA:(PL ​+ ​PLP), indicative of altered vitamin B6 homeostasis towards increased B6 catabolism. HK:XA and HK:XAA are indicative of increased HK in blood owing to reduction in the activity of the B6-dependent enzymes KAT and KYNU, respectively. HKr = HK: (KYNA ​+ ​XA ​+ ​HAA ​+ ​AA).

Abbreviations: AA, anthranilic acid; BMI, body-mass index; HAA, 3-hydroxyanthranilinic acid; HK, 3-hydroxykynurenine; KAT, kynurenine aminotransferase; KYNA, kynurenic acid; KYNU, kynureninase; NSAID, non-steroidal anti-inflammatory drug; PA, pyridoxic acid; PL, pyridoxal; PLP, pyridoxal 5′-phosphate; XA, xanthurenic acid.

a Adjusted for age, sex, BMI, smoking, education, presence of diabetes, presence of cardiovascular disease, use of NSAIDs.

Of our patient cohort, 31.9% had low to deficient plasma concentrations of PLP (<30 ​nmol/L) (Arevalo et al., 2019), though all of our controls had normal PLP concentrations. There were no differences noted between patients with low to deficient levels of PLP compared to those with normal levels with regard to age, sex, BMI, smoking, education, depression severity, depression subtype, presence of diabetes or cardiovascular disease, NSAID use, response to ECT, or the weight loss question (Item 5) on the HAMD-24 scale (Table 3). However, there was a significant difference with regard to reported family history of depression between the groups, with 60% of those with low to deficient PLP levels reporting a family history compared to 34.4% of those with normal PLP concentrations.

**Table 3:** Demographic and clinical characteristics of patients with low to deficient PLP concentrations compared to those with normal PLP concentrations.

	Low-to-deficient PLP Group (*n* ​= ​30)	Normal PLP Group (*n* ​= ​64)	Statistical test
Age, years	58.47 (12.10)	54.08 (15.67)	t ​= ​1.35, *p* ​= ​0.18
Sex, No. (%)			
Male	8 (26.67)	28 (43.75)	χ^2^ ​= ​2.52, *p* ​= ​0.11
Female	22 (73.33)	36 (56.25)	
BMI	26.37 (4.21)	26.95 (5.37)	t ​= ​−0.51, *p* ​= ​0.61
Smokers, No. (%)	14 (46.67)	26 (40.63)	χ^2^ ​= ​0.31, *p* ​= ​0.58
Education, No. (%)			
Primary	8 (26.67)	7 (10.94)	χ^2^ ​= ​5.09, *p* ​= ​0.08
Secondary	17 (56.67)	36 (56.25)	
Tertiary & Quaternary	5 (16.67)	21 (32.81)	
Bipolar depression, No. (%)	9 (30.00)	12 (18.75)	χ^2^ ​= ​1.49, *p* ​= ​0.22
Psychotic depression, No. (%)	8 (26.67)	13 (20.31)	χ^2^ ​= ​0.48, *p* ​= ​0.49
Diabetes, No. (%)	1 (3.33)	6 (9.38)	χ^2^ ​= ​1.08, *p* ​= ​0.30
Cardiovascular disease, No. (%)	14 (46.67)	25 (39)	χ^2^ ​= ​0.49, *p* ​= ​0.49
Baseline HAM-D24	30.93 (6.42)	31.05 (6.69)	t ​= ​−0.08, *p* ​= ​0.94
Post-ECT HAM-D24	10.10 (7.97)	10.60 (8.36)	t ​= ​−0.28, *p* ​= ​0.78
Number of ECT sessions	7.87 (2.19)	8.02 (2.59)	t ​= ​−0.27, *p* ​= ​0.79
Remitters, No. (%)	18 (60.00)	32 (50.00)	χ^2^ ​= ​0.82, *p* ​= ​0.37
Medications, No. (%) taking			
SSRI	9 (30.00)	13 (20.31)	χ^2^ ​= ​0.99, *p* ​= ​0.32
SNRI	13 (43.33)	35 (54.69)	χ^2^ ​= ​1.22, *p* ​= ​0.27
TCA	7 (23.33)	18 (28.13)	χ^2^ ​= ​0.28, *p* ​= ​0.59
MAOI	4 (13.33)	6 (9.38)	χ^2^ ​= ​0.31, *p* ​= ​0.58
Mirtazapine	8 (26.67)	24 (37.5)	χ^2^ ​= ​1.18, *p* ​= ​0.28
Bupropion	0 (0.00)	1 (1.56)	χ^2^ ​= ​0.48, *p* ​= ​0.49
Lithium	8 (26.67)	26 (40.63)	χ^2^ ​= ​1.87, *p* ​= ​0.17
Sodium Valproate/Lamotrigine	2 (6.67)	4 (6.25)	χ^2^ ​= ​0.003, *p* ​= ​0.95
Antipsychotics	25 (83.33)	40 (62.50)	χ^2^ ​= ​3.80, *p* ​= ​0.05
Benzodiazepines	17 (56.67)	32 (50.00)	χ^2^ ​= ​0.28, *p* ​= ​0.60
Non-benzodiazepine hypnotics	19 (63.33)	37 (57.81)	χ^2^ ​= ​0.18, *p* ​= ​0.67
Pregabalin	2 (6.67)	3 (4.69)	χ^2^ ​= ​0.15, *p* ​= ​0.70
Other	5 (16.67)	11 (17.19)	χ^2^ ​= ​0.009, *p* ​= ​0.92
NSAIDs	8 (26.67)	14 (21.88)	χ^2^ ​= ​0.26, *p* ​= ​0.61
HAMD-24 Item 5, No. (%)			χ^2^ ​= ​1.35, *p* ​= ​0.51
<1 lb weight loss in week	20 (66.67)	48 (75)	
>1 lb weight loss in week	5 (16.67)	8 (12.5)	
>2 lb weight loss in week	5 (16.67)	6 (9.38)	
Family history depression^a^			
Yes, No. (%)	18 (60.00)	22 (34.40)	χ^2^ ​= ​5.49, *p* ​= ​0.02

Data are presented as means with standard deviations (SD) or number (%) per group where appropriate.

Abbreviations: BMI, body mass index; ECT, electroconvulsive therapy; HAM-D24, Hamilton depression rating scale, 24-item version; MAOI, monoamine oxidase inhibitor; SNRI, serotonin-norepinephrine reuptake inhibitor; SSRI, selective serotonin reuptake inhibitor; TCA, tricyclic antidepressant.

a First or second degree relative.

#### 3.2.2 B vitamin concentrations in subgroups of patients with depression compared to controls

We subsequently conducted exploratory subgroup analyses to determine if there were any differences in B vitamin concentrations in patients with unipolar vs. bipolar depression (Supplemental Table 1) or psychotic vs. non-psychotic depression (Supplemental Table 2). The unadjusted analyses in Supplemental Table 1 show that flavin mononucleotide (B2) concentrations were significantly lower in patients with unipolar and bipolar depression compared to controls, and that concentrations were also lower in patients with bipolar compared to unipolar depression. Nicotinamide (B3), N1-methylnicotinamide (B3), PLP (B6), and PA (B6) concentrations also significantly differed in patients with unipolar or bipolar depression compared to controls, though there was no difference between depression subgroups. However, none of these results survived adjustment for potential confounders.

Flavin mononucleotide, N1-methylnicotinamide, PLP, and PL were all significantly lower in patients with either psychotic or non-psychotic depression compared to controls, though there was no difference between depressed subgroups (Supplemental Table 2). Nicotinamide was significantly lower in patients with non-psychotic depression compared to controls, and there was no difference between patients with psychotic depression vs. controls. Moreover, thiamine monophosphate (B1) was significantly lower in patients with psychotic depression compared to both healthy controls and patients with non-psychotic depression. Only PLP survived adjustment for potential confounders, though there was no difference in concentrations between the depression psychosis subgroups.

#### 3.2.3 Vitamin B function in patients with depression compared to controls

With regard to B vitamin function, the PAr (PA:(PL ​+ ​PLP)), HK:XA and HK:HAA ratios, and HKr (HK:(KYNA ​+ ​XA ​+ ​HAA ​+ ​AA) were significantly increased in patients with depression compared to controls (Table 2). As previously mentioned, an increased PAr index is indicative of altered vitamin B6 homeostasis towards increased B6 catabolism (Ueland et al., 2015). Moreover, increased HK:XA and HK:HAA ratios are indicative of increased HK in the plasma owing to a reduction in the activity of the B6-dependent enzymes KAT and KYNU, respectively (Ueland et al., 2015), while an increased HKr is indicative of decreased PLP activity (Ulvik et al., 2020). The PAr, HK:XA ratio, and HKr were also significantly increased in patients with unipolar and bipolar depression compared to controls; however, the HK:HAA ratio was significantly increased in patients with bipolar depression compared to controls and patients with unipolar depression (Supplemental Table 1). A significant difference was also noted in the HKr between the depressed subgroups. The PAr, HK:XA and HK:XAA ratios, and HKr were all significantly increased in patients with psychotic or non-psychotic depression compared to controls, but there was no difference between depressed subgroups (Supplemental Table 2).

### 3.3 Patients with depression pre- and post-ECT

#### 3.3.1 B vitamin concentrations and function in patients with depression pre- and post-ECT

Table 4 shows the plasma vitamin B concentrations from patients with depression assessed before and after (1–3 days) a course of ECT. Both unadjusted and adjusted analyses show that there was no change in vitamin B complex concentrations in patients post-ECT. Moreover, there was no difference in the PAr, HK:XA or HK:HAA ratios, or the HKr post-ECT.

**Table 4:** B vitamin plasma concentrations in medicated patients with depression before and following a course of ECT.

	Depressed Pre-ECT (*n* ​= ​94)	Depressed Post-ECT (*n* ​= ​94)	Cohen’s d	Unadjusted Statistics	Adjusted Statistics^a^
*B vitamins*					
Thiamine (B1)	5.80 (8.13)	6.02 (8.13)	0.03	*Z* ​= ​2467, *p* ​= ​0.377; *p*_FDR_ ​= ​0.85	
Thiamine Monophosphate (B1)	6.99 (2.80)	7.67 (3.11)	0.23	*Z* ​= ​2844.50, *p* ​= ​0.021; *p*_FDR_ ​= ​0.19	
Riboflavin (B2)	17.36 (17.87)	19.93 (27.55)	0.11	*Z* ​= ​2101, *p* ​= ​0.746; *p*_FDR_ ​= ​0.89	
Flavin Mononucleotide (B2)	9.99 (10.61)	9.58 (11.66)	−0.04	*Z* ​= ​1933, *p* ​= ​0.333; *p*_FDR_ ​= ​0.85	
Nicotinamide (B3)	968.59 (355.20)	985.90 (386.23)	0.05	*F*_1,93_ ​= ​0.246, *p* ​= ​0.621; *p*_FDR_ ​= ​0.89	*F*_1,81_ ​= ​0.40, *p* ​= ​0.53; *p*_FDR_ ​= ​0.53
N1-methylnicotinamide (B3)	115.06 (63.62)	111.24 (69.18)	−0.06	*F*_1,93_ ​= ​0.072, *p* ​= ​0.790; *p*_FDR_ ​= ​0.89	*F*_1,81_ ​= ​0.00006, *p* ​= ​0.99; *p*_FDR_ ​= ​0.53
Pyridoxal 5ʹ-phosphate (B6)	51.24 (50.71)	48.80 (39.18)	−0.05	*F*_1,93_ ​= ​0.000005, *p* ​= ​0.998; *p*_FDR_ ​= ​0.10	*F*_1,81_ ​= ​0.73, *p* ​= ​0.40; *p*_FDR_ ​= ​0.53
Pyridoxic Acid (B6)	37.09 (100.83)	32.81 (39.83)	−0.06	*Z* ​= ​2556, *p* ​= ​0.222; *p*_FDR_ ​= ​0.84	
Pyridoxal (B6)	18.40 (76.96)	11.89 (16.06)	−0.12	*Z* ​= ​2322.50, *p* ​= ​0.6; *p*_FDR_ ​= ​0.89	

*Ratios indicative of B vitamin function*					
PAr	0.54 (0.27)	0.56 (0.22)	0.08	Z ​= ​2381, *p* ​= ​0.58	
HK:XA	5.17 (3.23)	4.89 (3.52)	−0.08	Z ​= ​1923, *p* ​= ​0.24	
HK:HAA	1.45 (0.72)	1.39 (0.56)	0.09	Z ​= ​2128, *p* ​= ​0.69	
HKr	0.46 (0.18)	0.45 (0.15)	0.06	Z ​= ​2148, *p* ​= ​0.75	

Data are presented as mean (SD) nmol/L.

*p*_FDR_ represents the adjusted p-value following Benjamini-Hochberg analysis (only B vitamins were included in this analysis and not functional ratios).

PAr = PA:(PL ​+ ​PLP), indicative of altered vitamin B6 homeostasis towards increased B6 catabolism. HK:XA and HK:XAA are indicative of increased HK in blood owing to reduction in the activity of the B6-dependent enzymes KAT and KYNU, respectively. HKr = HK: (KYNA ​+ ​XA ​+ ​HAA ​+ ​AA).

Abbreviations: AA, anthranilic acid; BMI, body-mass index; HAA, 3-hydroxyanthranilinic acid; HK, 3-hydroxykynurenine; KAT, kynurenine aminotransferase; KYNA, kynurenic acid; KYNU, kynureninase; NSAID, non-steroidal anti-inflammatory drug; PA, pyridoxic acid; PL, pyridoxal; PLP, pyridoxal 5′-phosphate; XA, xanthurenic acid.

a Adjusted for age, sex, BMI, smoking, presence of diabetes, presence of cardiovascular disease, use of NSAIDs, and depression polarity, presence of psychosis, baseline depression severity where appropriate.

#### 3.3.2 B vitamin concentrations and function in depression subgroups pre- and post-ECT

Exploratory subgroup analyses were also conducted to determine if there was any difference between patients with unipolar vs. bipolar depression, psychotic vs. non-psychotic depression, or ECT remitters vs. non-remitters. We found no statistically significant difference between depression subgroups with regard to vitamin B concentrations, though there were numerical differences noted (Supplemental Tables 3–5). The HK:HAA ratio was significantly different between patients with unipolar and bipolar depression (Supplemental Table 3), indicative of a greater reduction in KYNU function in patients with bipolar disorder.

### 3.4 Associations with mood and clinical outcomes

Correlations between vitamin B complex concentrations and HAM-D24 scores were assessed before and after ECT. There were no significant associations between mood scores and any of the B vitamins in the depressed or control groups (data not shown). Moreover, no associations were identified in any of the depression subgroups (bipolar vs unipolar, psychotic vs. non-psychotic, remitters vs. non-remitters) (data not shown).

### 3.5 Associations with inflammatory mediators

**Fig. 2:**
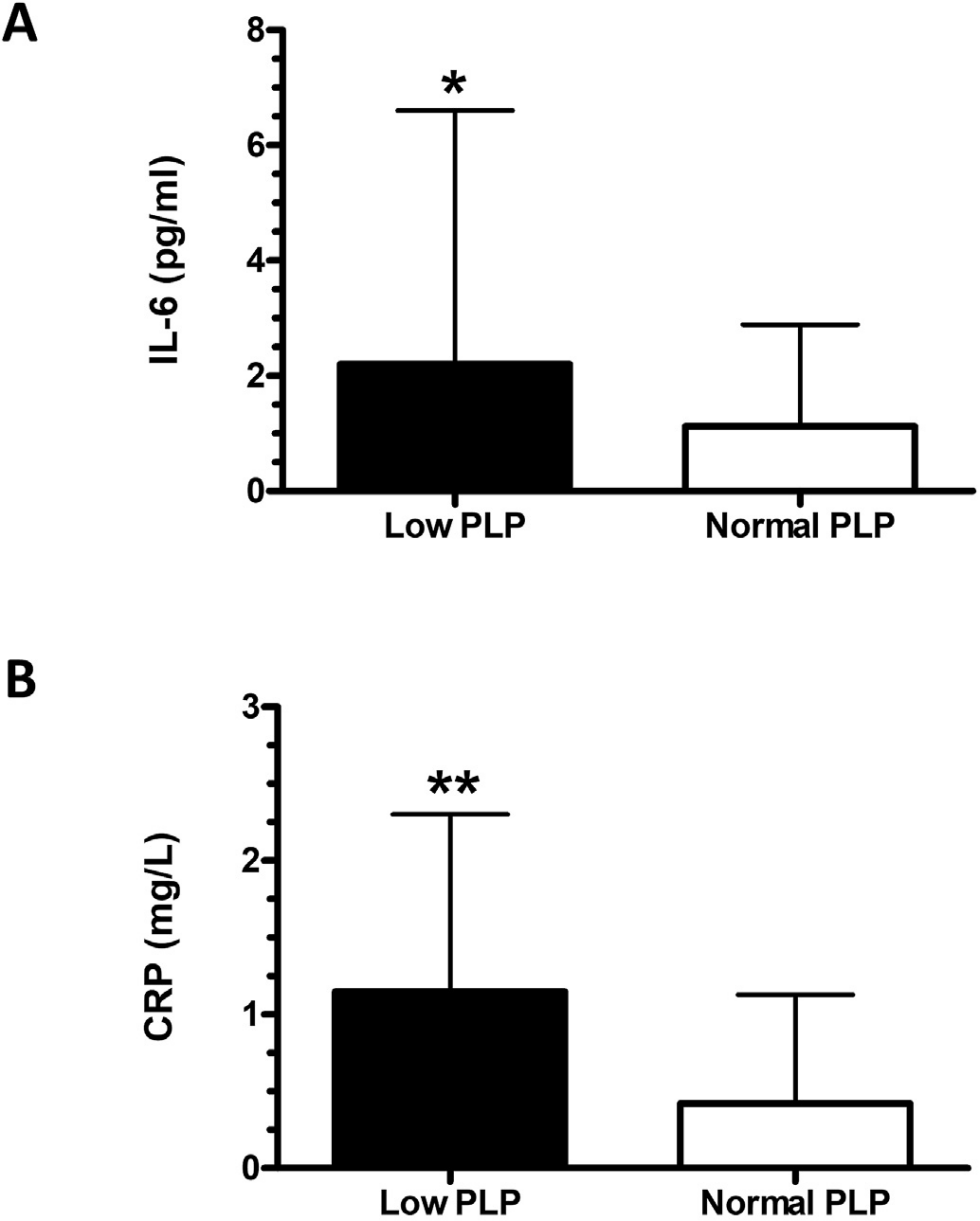
IL-6 and CRP concentrations in patients with depression with low to deficient plasma PLP concentrations compared to those with normal plasma concentrations. **(A)** IL-6 concentrations are displayed as mean ​+ SD for the group of patients with low to deficient (Low) plasma concentrations of PLP (vitamin B6) compared to those with normal (Normal) PLP concentrations. **(B)** CRP concentrations are displayed as mean ​+ SD for the group of patients with low to deficient (Low) plasma concentrations of PLP compared to those with normal (Normal) PLP concentrations. Low PLP: n ​= ​22; Normal PLP: n ​= ​46. ∗*p* ​< ​0.05, ∗∗*p* ​< ​0.01 vs Normal PLP.

We next assessed if there was any relationship between PLP (B6), nicotinamide (B3), or N1-methylnicotinamide (B3) concentrations and concentrations of inflammatory mediators (IL-6, IL-10, TNF-α, or CRP) at baseline or following a course of ECT. There was a significant negative correlation between PLP and IL-6 (*ρ* ​= ​−0.39, *p* ​= ​0.001) and CRP (*ρ* ​= ​−0.45, *p* ​= ​0.00003) at baseline, with patients with low to deficient concentrations (<30 ​nmol/L) of PLP having the highest concentrations of IL-6 and CRP (Fig. 2A and B). This finding was specific to IL-6 and CRP since no correlation was identified between PLP and IL-10 or TNF-α (*ρ* ​= ​−0.06, *p* ​= ​0.61 and *ρ* ​= ​−0.12, *p* ​= ​0.30, respectively). Interestingly, the change in nicotinamide (B3) following a course of ECT was positively correlated with the change in TNF-α (*r* ​= ​0.32, *p* ​= ​0.004). No other significant associations were identified.

### 3.6 Associations with tryptophan and kynurenine pathway metabolites

We subsequently assessed if there was any relationship between PLP (B6), nicotinamide (B3), or N1-methylnicotinamide (B3) concentrations and concentrations of tryptophan or metabolites of the kynurenine pathway. We identified a significant moderate positive relationship between PLP and tryptophan concentrations at baseline (ρ ​= ​0.41, *p* ​= ​0.0004) and the change in PLP and change in tryptophan following a course of ECT (ρ ​= ​0.37, *p* ​= ​0.0003). There was also a significant positive relationship between the KYN/TRP ratio, indicative of cellular immune activation and activation of the TRP-degrading enzyme indoleamine 2,3-dioxygenase (Ulvik et al., 2016), and PA (B6 catabolite) at baseline in patients with depression (ρ ​= ​0.35, p ​= ​0.001), as well as between KYN/TRP and the PAr (ρ ​= ​0.301, p ​= ​0.003), and a negative relationship between PLP and KYN/TRP (ρ ​= ​−0.26, *p* ​= ​0.01) and between nicotinamide and KYN/TRP (ρ ​= ​−0.30, *p* ​= ​0.003). In addition, there was a significant association between the change in KYN/TRP and change in PA concentration post-ECT (ρ ​= ​0.30, p ​= ​0.003). No other significant associations were noted.

## 4 Discussion

To our knowledge, the present study is the first to assess the role of vitamin B plasma concentrations in the response to ECT for depression. Our results show that circulating plasma concentrations of PLP (vitamin B6), nicotinamide (vitamin B3), and N1-methylnicotinamide (vitamin B3) are significantly reduced on average by 36, 14, and 23%, respectively, in patients with depression compared to healthy controls after adjusting for potential confounders and multiple comparisons. These results are in line with the extant literature on B vitamins in depression and show a specific pattern of changes in B vitamins in medicated patients with depression, as no significant changes were noted in other B vitamins analyzed. However, plasma vitamin B concentrations were unaltered by treatment with ECT, and our exploratory analyses showed there was no significant difference in vitamin B concentrations between patients with unipolar vs. bipolar depression or psychotic vs. non-psychotic depression. There was also no difference in vitamin B concentrations in ECT remitters vs. non-remitters, thus refuting our hypothesis that ECT remitters would have higher vitamin B concentrations. Moreover, in contrast to reports from others (Arevalo et al., 2019; Hvas et al., 2004; Moore et al., 2019), vitamin B concentrations were not associated with depression symptom severity. However, in line with reports in other medical conditions (see Ueland et al., 2017 for review), our exploratory analyses found that PLP concentrations were negatively associated with IL-6 and CRP concentrations at baseline in patients with depression, while, interestingly, the change in nicotinamide (vitamin B3) following a course of treatment with ECT was positively associated with the change in TNF-α. In addition, a significant relationship was noted between plasma PLP and tryptophan concentrations both at baseline and following ECT, while relationships between B6 vitamins and the tryptophan-kynurenine pathway were also identified.

PLP, the active form of vitamin B6, is a co-factor in over 150 enzymatic reactions, constituting about 4% of all enzymatic activities, including amino acid synthesis, one carbon metabolism, and neurotransmitter biosynthesis (Ueland et al., 2017). One study conducted in the USA found that 10.5% of the general population (*n* ​= ​8311) were deficient in PLP (CDC, 2012); thus, our finding that 31.9% of patients with depression had low to deficient levels of PLP is about three times the figure reported in the general population. Our results are in line with those of Bell et al. (1991) who showed that 28% of young adult (*n* ​= ​16) or geriatric inpatients (*n* ​= ​20) with major depression were deficient in vitamins B2, B6, and B12, and this percentage did not differ between the age groups. We also found that the percentage of patients with depression with low to deficient concentrations of PLP did not differ between groups aged under 60 years or ≥60 years (data not shown).

In contrast to previous reports, we found no association between plasma PLP (B6) concentrations and mood in our severely depressed cohort, either in the group as a whole or when we divided the group into those with normal concentrations of PLP vs. those with low to deficient concentrations. In a large (*n* ​= ​5186) cross-sectional study of an Irish community-dwelling sample aged ≥60 years, B2, B6, and B9 levels in the lowest quintile were each associated with an increased risk for depressive symptoms, and B vitamin fortified foods were found to be associated with a decreased risk of depression if consumed daily (Moore et al., 2019). Plasma PLP concentrations were also found to be associated with depressive symptoms over 5–7 years in a population-based cohort of older Puerto Rican adults residing in northeastern USA (*n* ​= ​1446), and those participants who had suboptimal PLP concentrations (<30 ​mg/dl) had higher baseline depressive symptoms compared to participants with normal levels, a finding that persisted over time and withstood adjustment for numerous potential confounders (Arevalo et al., 2019). A further study showed that low plasma PLP levels were significantly associated with depression symptom severity in an elderly Danish community sample (mean age 75 years; *n* ​= ​140); those with PLP concentrations below 3 ​nmoL/l reporting more frequent symptoms of depression (Hvas et al., 2004). Moreover, Skarupski et al. (2010) showed that higher total dietary intakes of vitamins B6, B9, and B12 were associated with a decreased likelihood of developing depression in a study of older adults in the USA (*n* ​= ​3503) followed-up for an average of 7.2 years. Of note, to our knowledge, no study of clinically diagnosed patients with depression has found an association between mood and PLP status. A limitation of our study is that dietary information regarding vitamin B intake was unavailable; however, there were no differences noted between patients with low-deficient PLP compared to those with normal levels regarding demographic or clinical variables, including the weight loss item on the HAM-D24 scale. In spite of this, it may be the case that patients with depression favor a diet that is low in vitamin B and thus further studies are required to rule this out. Notably, more patients with low-deficient plasma concentrations of PLP had a family history of depression than those with normal PLP concentrations, suggesting a role for heritable or environmental factors in these changes, though this requires further investigation.

In addition to the difference in PLP concentrations, our results showed reduced plasma concentrations of nicotinamide (B3) and its metabolite N1-methylnicotinamide in patients with depression compared to healthy controls. There has been a limited number of studies examining circulating nicotinamide concentrations and depression. In line with our results, a recent study by Colle et al. (2019) showed that nicotinamide concentrations were lower in depressed patients (*n* ​= ​173) compared to healthy controls (*n* ​= ​214). Notably, 35% of the patients with depression in that study were antidepressant-free for 3 years and so the authors were able to show that nicotinamide levels were not associated with antidepressant status, thus reducing the likelihood that our results are influenced by antidepressant medications.

Nicotinamide, the amide form of vitamin B3, plays an important role in neuronal differentiation, survival, and function, and has been indirectly implicated in neuroprotection through inhibition of apoptosis (Fricker et al., 2018; Song et al., 2019). It also plays a role in maintenance of DNA integrity and cell membrane asymmetry to prevent cellular inflammation, phagocytosis, and vascular thrombosis; modulates Akt signaling, caspase activity, and energy metabolism; and provides robust cytoprotection (Maiese and Chong, 2003). Nicotinamide undergoes degradation by nicotinamide N-methyltransferase to N1-methylnicotinamide, which itself may function as an anti-thrombotic, anti-inflammatory, and liver protection agent (Song et al., 2019). Thus, the reduced nicotinamide concentrations observed in our patient cohort could have a wide-ranging impact. Once incorporated into cells, nicotinamide can be converted via the salvage pathway to nicotinamide adenine dinucleotide (NAD+), a co-factor or substrate for approximately 500 cellular reactions (Klimova and Kristan, 2019).

Plasma PLP is suggested to reflect vitamin B6 status in the liver, which contains a rapidly exchanging PLP pool that is mobilized to sites of inflammation via the blood (Ulvik et al., 2012). An array of studies have reported PLP concentrations to be low in conditions with an inflammatory component such as rheumatoid arthritis, inflammatory bowel disease, and diabetes (see Ueland et al., 2017 for review). In this regard, plasma PLP has been shown to be inversely related to CRP concentrations, IL-6R levels, and the KYN/TRP ratio (Midttun et al., 2011; Theofylaktopoulou et al., 2014; Ulvik et al., 2012). In line with these reports, our results show that PLP inversely correlated with IL-6 and CRP levels and the KYN/TRP ratio, and positively correlated with tryptophan concentrations. Notably, both IL-6 and CRP levels were found to be higher in those patients with low to deficient levels of PLP compared to those with normal levels. We also showed that PA (vitamin B6 catabolite) was positively related to the KYN/TRP ratio, again in line with a previous report (Ulvik et al., 2012). It has been suggested that, owing to the impact of IL-6 on hepatocytes, IL-6 may function in activating PLP phosphatase activity, thus decreasing circulating PLP concentrations (McCarty, 2000), though this remains to be assessed in patients with depression. Most previous studies that have shown a link between low PLP concentrations and inflammatory conditions have noted that there is no indication of low dietary intake of vitamin B6 (Paul et al., 2013) or excessive excretion (Chiang et al., 2005). Instead, it appears that inflammation causes tissue-specific depletion of vitamin B6. For example, in a study of patients with rheumatoid arthritis, inflammation was associated with low plasma PLP concentrations but normal erythrocyte PLP concentrations and urinary PA excretion, and in the same study the authors showed that, in an animal model of arthritis, inflammation was associated with lower circulating and liver PLP concentrations but normal PA excretion and PLP storage in muscle (Chiang et al., 2005).

Our results also show that the PAr was increased in patients with depression compared to controls, indicative of increased B6 catabolism owing to inflammation (Ueland et al., 2015). Furthermore, in line with previous reports (Ulvik et al., 2016), the PAr significantly correlated with the inflammatory markers CRP (acute phase marker) and KYN/TRP (cellular immunity marker) in our depressed cohort. A previous *in vitro* study showed that nicotinamide can reduce endotoxin-induced production of the pro-inflammatory cytokines IL-6 or TNF-α in human whole blood (Ungerstedt et al., 2003); however, we found no associations between nicotinamide and IL-6 though, interestingly, our results show that the change in nicotinamide levels positively correlated with the change in TNF-α levels following a course of ECT. We have previously shown TNF-α to be implicated in activation of the kynurenine pathway post-ECT (Ryan et al., 2020).

Low plasma PLP does not necessarily indicate intracellular vitamin B6 deficiency, since vitamins can become redistributed into the intercellular space during inflammation, and so the measurement of functional indicators, such as the HK:XA and HK:HAA ratios and HKr, is also required (Ulvik et al., 2013, 2020). We found that the HK:XA and HK:HAA ratios were increased in depressed patients compared to controls, indicative of an increase in the kynurenine pathway metabolite HK in the plasma owing to a reduction in function of the B6-dependent enzymes KAT and KYNU, respectively, while the HKr was also increased (see Fig. 1 for reference). Further studies are needed to fully elucidate if the low plasma PLP concentrations observed in patients with depression are associated with low dietary intake of vitamin B6, increased excretion, or some other cause.

While plasma levels of other B vitamins examined in this study did not significantly differ between depressed patients and controls, this may not be the case at a central level. A study by Pan et al. (2017) recently showed that though serum levels of folate were normal in a small cohort of young adults with treatment-resistant depression compared to healthy controls, 36% of the patient cohort had low cerebrospinal fluid 5-methyltetrahydrofolate levels, and treatment with folinic acid improved depressive symptoms in all patients who completed the treatment course. However, a strong correlation has been shown between PLP (active B6), PL (B6 transport form), and PA (B6 catabolite) concentrations in cerebrospinal fluid and in plasma, with the strongest correlation found for PLP (Albersen et al., 2014). Thus, it is likely that the low PLP concentrations observed in depressed patients in our study are also reflected in, and have impacts on, the brain. The impact of other B vitamins should not be ruled out entirely until further investigations on their peripheral and central concentrations have been conducted.

The reason for the low circulating concentrations of PLP (B6), nicotinamide (B3), and N1-methylnicotinamide (B3) observed in depressed patients in our study are unclear. It has been suggested that patients with depression lack the ability to metabolize B vitamins owing to the presence of genetic polymorphisms that result in decreased vitamin metabolism (Mech and Farah, 2016). Changes in the gut microbiota that have been reported in depression (Jiang et al., 2015) may also be a contributory factor since B vitamins are synthesized by various gut commensals (Yoshii et al., 2019) in addition to being consumed directly through the diet. A further possibility is that patients with depression have an impaired ability to absorb B vitamins, since absorption of vitamins B3 and B6 occurs directly through the small intestine (Mikkelsen et al., 2016; Yoshii et al., 2019). Though the evidence for this is weak so far, if there is in fact a reduced ability to absorb or metabolize B vitamins in depression, one way to circumvent this may be via oral administration of reduced forms of B vitamins or direct intravenous/intramuscular injection. Thus, further studies are required to determine the exact cause of reduced circulating vitamin B concentrations in depressed patients and whether this is also reflected in the brain.

A strength of our study was that strict criteria were used for diagnosing depression as part of a clinical trial, which only included patients with a robust phenotype of severe depression referred for ECT. Moreover, the trial sample was representative of the typical depressed population referred for ECT (Semkovska et al., 2016). Another strength is that our study is the first to examine B vitamins in the response to ECT in a real-world clinical setting. In addition, we assessed a panel of B vitamins, which included some lesser-studied B vitamins in depression, and our results show specificity to changes in vitamins B3 and B6. Thus, as changes were not noted across the entire panel examined, the results reported are unlikely to be due to diet alone.

There are also several limitations to our study. First, all of our patients were receiving treatment with pharmacotherapy-as-usual during the course of ECT. Therefore, the low PLP (B6), nicotinamide (B3), and N1-methylnicotinamide (B3) concentrations observed in our patient sample at baseline compared to controls might be accounted for by the effects of psychotropic drugs since it has been shown that certain drugs can have inhibitory effects on pyridoxal kinase, which is involved in the conversion of PL to PLP, thereby inducing PLP deficiency (di Salvo et al., 2011). However, as already discussed, the study by Colle et al. (2019) suggests that the reduction in nicotinamide concentrations is not due to antidepressant drugs; further study is required to assess this in full. Second, we did not have data available on all components of the vitamin B complex, only those determined using LC/MS as part of the panel assessed for our previous study (Ryan et al., 2020). Therefore, future studies should aim to examine the vitamin B complex in its entirety in depression. Third, as already discussed, we did not have information available on dietary intake of B vitamins or use of vitamin supplements.

Overall, our findings of lower peripheral blood concentrations of vitamins B6 (PLP) and B3 (nicotinamide and N1-methylnicotinamide) in patients with depression could have wide-ranging effects on pathways and systems previously implicated in depression. Further studies are required to understand the reasons why patients present with these low B vitamin concentrations, which seems to be unlikely due to dietary insufficiency but could be due to a problem with absorption from the gut, and whether treatment with various forms of B vitamin supplements has antidepressant effects.

## Declaration of competing interest

DMM has received speaker’s honoraria from MECTA and Otsuk and an honorarium from Janssen for participating in an esketamine advisory board meeting. KAA is employed by Boehringer Ingelheim Pharma GmbH & Co. KG. KR and AH have no interests to declare. This work was supported by the Health Research Board (HRB), Ireland (TRA/2007/5 & HPF/2010/17).
