# Supplementary material for: Blood plasma B vitamins in depression and the therapeutic response to electroconvulsive therapy
Source: Brain Behav Immun Health. 2020 Mar 28;4:100063. doi: 10.1016/j.bbih.2020.100063 (PMC8474603; doi:10.1016/j.bbih.2020.100063)
Supplement: Multimedia component 1 [file mmc1.docx]

|  | **Control (*n*=57)** | **Unipolar (*n*=73)** | **Bipolar (*n*=21)** | **Statistics** | **Adjusted Statistics^#^** |
| --- | --- | --- | --- | --- | --- |
| *B vitamins* |  |  |  |  |  |
| Thiamine (B1) | 4.25 (2.01) | 6.29 (9.15) | 4.10 (1.38) | *F*_2,148_ = 1.91, *p* = 0.15, *p*_FDR_ = 0.19 | *F*_2,131_ = 0.94, *p* = 0.39, *p*_FDR_ = 0.39 |
| Thiamine Monophosphate (B1) | 7.50 (2.24) | 7.14 (3.05) | 6.48 (1.67) | *F*_2,148_ = 1.61, *p* = 0.20, *p*_FDR_ = 0.23 | *F*_2,131_ = 0.94, *p* = 0.39, *p*_FDR_ = 0.39 |
| Riboflavin (B2) | 18.63 (16.55) | 17.78 (19.34) | 15.92 (11.64) | H = 0.732, *p* = 0.69, *p*_FDR_ = 0.61 |  |
| Flavin Mononucleotide (B2) | 13.27 (12.77) | 10.70 (11.87)** | 7.54 (2.88)** + | H = 16.15, *p* < 0.001, ***p*_FDR_ = 0.003** |  |
| Nicotinamide (B3) | 1126.58 (314.31) | 976.51 (362.11)* | 941.05 (337.04)* | *F*_2,148_ = 3.89, *p* = 0.02, ***p*_FDR_ = 0.04** | *F*_2,131_ = 2.67, *p* = 0.07, *p*_FDR_ = 0.14 |
| N1-methylnicotinamide (B3) | 149.92 (73.41) | 118.21 (64)* | 104.11 (62.55)* | *F*_2,148_ = 5.98, *p* = 0.003, ***p*_FDR_ = 0.007** | *F*_2,131_ = 3.52, *p* = 0.03, *p*_FDR_ = 0.12 |
| Pyridoxal 5ʹ-phosphate (B6) | 79.90 (46.56) | 54.76 (55.80)** | 39.01 (23.37)** | H = 38.84, *p* < 0.001, ***p*_FDR_ = 0.003** |  |
| Pyridoxic Acid (B6) | 32.70 (15.63) | 40.73 (114.23) | 24.43 (9.39)* | H = 5.84, *p* = 0.05, ***p*_FDR_** = **0.003** |  |
| Pyridoxal (B6) | 16.68 (11.15) | 21.36 (87.23) | 8.12 (2.15) | H = 42.75, *p* < 0.001, *p*_FDR_ = 0.08 |  |
|  |  |  |  |  |  |
| *Ratios indicative of B vitamin function* | | | | | |
| PAr | 0.37 (0.14) | 0.53 (0.25)** | 0.60 (0.31)** | H = 21.05, *p* < 0.000003 |  |
| HK:XA | 2.69 (1.46) | 4.92 (2.99)** | 6.02 (3.91)** | H = 43.05, *p* = 4.49 ×10^-10^ |  |
| HK:HAA | 1.13 (0.32) | 1.34 (0.63) | 1.82 (0.90)** + | H = 13.23, *p =* 0.001 |  |
| HKr | 0.34 (0.08) | 0.44 (0.17)** | 0.54 (0.19)**+ | H = 28.02, *p* = 8.24 × 10^-7^ |  |

| **Supplemental Table 1. B vitamins in controls compared to patients with unipolar or bipolar depression** |
| --- |

Data are presented as mean (SD) nmol/L.

**p* < 0.05, ** *p* < 0.001 vs control.

+*p* < 0.05 vs unipolar group.

*p*_FDR_ represents the adjusted p-value following Benjamini-Hochberg analysis (only B vitamins were included in this analysis and not functional ratios).  *p*_FDR_ highlighted in bold attains statistical significance.

^#^ adjusted for age, sex, BMI, smoking, presence of diabetes, presence of cardiovascular disease, use of NSAIDs.

PAr = PA:(PL + PLP), indicative of altered vitamin B6 homeostasis towards increased B6 catabolism. HK:XA and HK:HAA are indicative of increased HK in blood owing to reduction in the activity of the B6-dependent enzymes KAT and KYNU, respectively. HKr = HK: (KYNA + XA + HAA + AA).

Abbreviations: AA, anthranilic acid; BMI, body-mass index; HAA, 3-hydroxyanthranilinic acid; HK, 3-hydroxykynurenine; KAT, kynurenine aminotransferase; KYNA, kynurenic acid; KYNU, kynureninase; NSAID, non-steroidal anti-inflammatory drug; PA, pyridoxic acid; PL, pyridoxal; PLP, pyridoxal 5′-phosphate; XA, xanthurenic acid.
