# Supplementary material for: Blood plasma B vitamins in depression and the therapeutic response to electroconvulsive therapy
Source: Brain Behav Immun Health. 2020 Mar 28;4:100063. doi: 10.1016/j.bbih.2020.100063 (PMC8474603; doi:10.1016/j.bbih.2020.100063)
Supplement: Multimedia component 2 [file mmc2.docx]

| **Supplemental Table 2. B vitamin in controls compared to patients with psychotic or non-psychotic depression** | | | | | | |  |
| --- | --- | --- | --- | --- | --- | --- | --- |
|  | **Control**  **(*n*=57)** | **Psychotic (*n*=21)** | **Non-psychotic (*n*=73)** | **Statistics** | | | **Adjusted Statistics^#^** |
|  |  |  |  | |  | |  |
| *B vitamins* |  |  |  | |  | |  |
| Thiamine (B1) | 4.25 (2.01) | 3.65 (1.63) | 6.41 (9.11) | *F*_2,148_ = 2.48, *p* = 0.09, *p*_FDR_ = 0.09 | | *F*_2,131_ = 2.95, *p* = 0.06, *p*_FDR_ = 0.075 | |
| Thiamine Monophosphate (B1) | 7.50 (2.24) | 6.01 (2.43) * + | 7.27 (2.85) | *F*_2,148_ = 4.34, *p* = 0.02, ***p*_FDR_ = 0.03** | | *F*_2,131_ = 2.95, *p* = 0.06, *p*_FDR_ = 0.075 | |
| Riboflavin (B2) | 18.63 (16.55) | 22.20 (32.71) | 15.97 (10.33) | H = 0.795, *p* = 0.672, *p*_FDR_ = 0.60 | |  | |
| Flavin Mononucleotide (B2) | 13.27 (12.77) | 12.97 (21.19)* | 9.14 (4.16)** | H = 12.22, *p* = 0.002, ***p*_FDR_ = 0.005** | |  | |
| Nicotinamide (B3) | 1126.58 (314.31) | 961.69 (331.18) | 970.57 (363.98)* | *F*_2,148_ = 3.80, *p* = 0.025, ***p*_FDR_ = 0.03** | | *F*_2,131_ = 2.38, *p* = 0.10, *p*_FDR_ = 0.1 | |
| N1-methylnicotinamide (B3) | 149.92 (73.41) | 106.75 (65.56)* | 117.45 (63.31)* | *F*_2,148_ = 5.82, *p* = 0.004, ***p*_FDR_ = 0.008** | | *F*_2,131_ = 3.23, *p* = 0.04, *p*_FDR_ = 0.075 | |
| Pyridoxal 5ʹ-phosphate (B6) | 79.90 (46.56) | 43.20 (29.93)* | 53.55 (55.22)* | *F*_2,148_ = 18.33, *p* = 7.72×10^-8^, ***p*_FDR_ = 0.004** | | *F*_2,131_ = 7.60, *p* = 0.001, ***p*_FDR_ = 0.005** | |
| Pyridoxic Acid (B6) | 32.70 (15.63) | 24.69 (12.37) | 40.65 (114.16) | H = 6.20, *p* = 0.05, ***p*_FDR_ = 0.004** | |  | |
| Pyridoxal (B6) | 16.68 (11.15) | 9.66 (4.41)** | 20.91 (87.27)** | H = 39.95, *p* < 0.001, *p*_FDR_ = 0.06 | |  | |
|  |  |  |  |  | |  | |
| *Ratios indicative of B vitamin function* | | | | | | | |
| PAr | 0.37 (0.14) | 0.53 (0.24)** | 0.55 (0.27)** | H = 20.40, *p* < 0.00004 | |  | |
| HK:XA | 2.69 (1.46) | 6.32 (4.76)** | 4.84 (2.58)** | H = 42.80, *p* = 5.09 × 10^-10^ | |  | |
| HK:HAA | 1.13 (0.32) | 1.75 (1.07)* | 1.36 (0.57)* | H = 8.38, *p* < 0.02 | |  | |
| HKr | 0.34 (0.08) | 0.53 (0.25)** | 0.45 (0.15)** | H = 25.06, *p* = 0.000004 | |  | |

Data are presented as mean (SD) nmol/L.

**p* < 0.05, ** *p* < 0.001 vs control.

+*p* < 0.05 vs psychotic group.

*p*_FDR_ represents the adjusted p-value following Benjamini-Hochberg analysis (only B vitamins were included in this analysis and not functional ratios).  *p*_FDR_ highlighted in bold attains statistical significance.

^#^ adjusted for age, sex, BMI, smoking, presence of diabetes, presence of cardiovascular disease, use of NSAIDs.

PAr = PA:(PL + PLP), indicative of altered vitamin B6 homeostasis towards increased B6 catabolism. HK:XA and HK:XAA are indicative of increased HK in blood owing to reduction in the activity of the B6-dependent enzymes KAT and KYNU, respectively. HKr = HK: (KYNA + XA + HAA + AA). Abbreviations: AA, anthranilic acid; BMI, body-mass index; HAA, 3-hydroxyanthranilinic acid; HK, 3-hydroxykynurenine; KAT, kynurenine aminotransferase; KYNA, kynurenic acid; KYNU, kynureninase; NSAID, non-steroidal anti-inflammatory drug; PA, pyridoxic acid; PL, pyridoxal; PLP, pyridoxal 5′-phosphate; XA, xanthurenic acid.
