# Supplementary material for: Blood plasma B vitamins in depression and the therapeutic response to electroconvulsive therapy
Source: Brain Behav Immun Health. 2020 Mar 28;4:100063. doi: 10.1016/j.bbih.2020.100063 (PMC8474603; doi:10.1016/j.bbih.2020.100063)
Supplement: Multimedia component 3 [file mmc3.docx]

| **Supplemental Table 3**  B vitamin plasma concentrations in patients with unipolar or bipolar depression pre- and post-ECT | | | | | |
| --- | --- | --- | --- | --- | --- |
|  | **Polarity** | **Pre-ECT** | **Post-ECT** | **Unadjusted Statistics** | **Adjusted Statistics^#^** |
| *B vitamins* |  |  |  |  |  |
| Thiamine (B1) | Unipolar | 6.29 (9.15) | 6.55 (9.12) | *Pre-ECT: U* = 729.50, *p* = 0.74  *Post-ECT: U =* 647, *p =* 0.28  *Unipolar: Z =* 1530.50, *p =* 0.32  *Bipolar: Z =* 115, *p =* 0.99 |  |
|  | Bipolar | 4.10 (1.38) | 4.18 (1.76) |  |  |
|  | *Cohen’s d* | *0.33* | *0.36* |  |  |
|  |  |  |  |  |  |
| Thiamine Monophosphate (B1) | Unipolar | 7.14 (3.05) | 7.94 (3.33) | *Pre-ECT: U* = 722, *p* = 0.69  *Post-ECT: U =* 612.50, *p =* 0.16  *Unipolar: Z =* 1794.50, *p =* 0.02  *Bipolar: Z =* 122, *p =* 0.82 |  |
|  | Bipolar | 6.48 (1.67) | 6.74 (1.96) |  |  |
|  | *Cohen’s d* | *0.27* | *0.44* |  |  |
|  |  |  |  |  |  |
| Riboflavin (B2) | Unipolar | 17.78 (19.34) | 21.05 (30.65) | *Pre-ECT: U* = 753.50, *p* = 0.91  *Post-ECT: U =* 809.50, *p =* 0.70  *Unipolar: Z =* 1242, *p =* 0.69  *Bipolar: Z =* 115.50, *p =* 1.00 |  |
|  | Bipolar | 15.92 (11.64) | 16.04 (11.32) |  |  |
|  | *Cohen’s d* | *0.12* | *0.22* |  |  |
|  |  |  |  |  |  |
| Flavin Mononucleotide (B2) | Unipolar | 10.70 (11.87) | 10.05 (13.14) | *Pre-ECT: U* = 532.50, *p* = 0.034  *Post-ECT: U =* 729.50, *p =* 0.74  *Unipolar: Z =* 985.50, *p =* 0.07  *Bipolar: Z =* 156, *p =* 0.16 |  |
|  | Bipolar | 7.54 (2.88) | 7.97 (2.63) |  |  |
|  | *Cohen’s d* | *0.37* | *0.22* |  |  |
|  |  |  |  |  |  |
|  |  |  |  |  |  |
| Nicotinamide (B3) | Unipolar | 976.51 (362.11) | 1001.68 (390.59) | *Time: F*_1,92_ = 0.03, *p* = 0.87  *Group: F*_1,92_ = 0.36, *p* = 0.55  *Group×Time: F*_1,92_ = 0.21, *p* = 0.65 | *Time: F*_1,81_ = 0.40, *p* = 0.53  *Group: F*_1,81_ = 0.49, *p* = 0.49  *Group×Time: F*_1,81_ = 0.26, *p* = 0.61 |
|  | Bipolar | 941.05 (337.04) | 931.06 (374.67) |  |  |
|  | *Cohen’s d* | *0.10* | *0.18* |  |  |
|  |  |  |  |  |  |
| N1-methylnicotinamide (B3) | Unipolar | 118.21 (64) | 111.72 (73.51) | *Time: F*_1,92_ = 0.21, *p* = 0.65  *Group: F*_1,92_ = 0.35, *p* = 0.56  *Group×Time: F*_1,92_ = 1.53, *p* = 0.22 | *Time: F*_1,81_ = 0.00006, *p* = 0.99  *Group: F*_1,81_ = 0.28, *p* = 0.60  *Group×Time:* *F*_1,81_ = 2.88, *p* = 0.09 |
|  | Bipolar | 104.11 (62.55) | 109.59 (52.89) |  |  |
|  | *Cohen’s d* | *0.22* | 0.03 |  |  |
|  |  |  |  |  |  |
| Pyridoxal 5ʹ-phosphate (B6) | Unipolar | 54.76 (55.80) | 52.10 (43.40) | *Pre-ECT: U* = 568, *p* = 0.07  *Post-ECT: U =* 629, *p =* 0.21  *Unipolar: Z =* 1294, *p =* 0.76  *Bipolar: Z =* 143, *p =* 0.34 |  |
|  | Bipolar | 39.01 (23.37) | 37.34 (13.49) |  |  |
|  | *Cohen’s d* | *0.37* | *0.46* |  |  |
|  |  |  |  |  |  |
| Pyridoxic Acid (B6) | Unipolar | 40.73 (114.23) | 35.06 (44.63) | *Pre-ECT: U* = 687, *p* = 0.47  *Post-ECT: U =* 616.50, *p =* 0.17  *Unipolar: Z =* 1566.50, *p =* 0.24  *Bipolar: Z =* 123*, p =* 0.79 |  |
|  | Bipolar | 24.43 (9.39) | 25.01 (11.03) |  |  |
|  | *Cohen’s d* | *0.20* | *0.31* |  |  |
|  |  |  |  |  |  |
| Pyridoxal (B6) | Unipolar | 21.36 (87.23) | 12.73 (18.11) | *Pre-ECT: U* = 583.50, *p* = 0.10  *Post-ECT: U =* 711*, p =* 0.61  *Unipolar: Z =* 1313, *p =* 0.10  *Bipolar: Z =* 148, *p =* 0.26 |  |
|  | Bipolar | 8.12 (2.15) | 8.95 (2.68) |  |  |
|  | *Cohen’s d* | *0.21* | *0.29* |  |  |
|  |  |  |  |  |  |
| *Ratios indicative of B vitamin function* | | | | | |
| PAr | Unipolar | 0.53 (0.25) | 0.56 (0.23) | *Pre-ECT: U* = 869, *p* = 0.35  *Post-ECT: U =* 827*, p =* 0.58  *Unipolar: Z =* 1573, *p =* 0.22  *Bipolar: Z =* 85*, p =* 0.29 |  |
|  | Bipolar | 0.60 (0.31) | 0.56 (0.21) |  |  |
|  | *Cohen’s d* | *0.25* | *0.00* |  |  |
|  |  |  |  |  |  |
|  |  |  |  |  |  |
| HK:XA | Unipolar | 4.92 (2.99) | 4.87 (3.80) | *Pre-ECT: U* = 888, *p* = 0.27  *Post-ECT: U =* 860*, p =* 0.40  *Unipolar: Z =* 1238, *p =* 0.54  *Bipolar: Z =* 78*, p =* 0.19 |  |
|  | Bipolar | 6.02 (3.91) | 4.95 (2.37) |  |  |
|  | *Cohen’s d* | *0.32* | *0.03* |  |  |
|  |  |  |  |  |  |
|  |  |  |  |  |  |
|  |  |  |  |  |  |
| HK:HAA | Unipolar | 1.34 (0.63) | 1.31 (0.53) | *Pre-ECT: U* = 1045, *p* = 0.01  *Post-ECT: U =* 1058*, p =* 0.008  *Unipolar: Z =* 1395, *p =* 0.81  *Bipolar: Z =* 127*, p =* 0.69 |  |
|  | Bipolar | 1.82 (0.90) | 1.67 (0.57) |  |  |
|  | *Cohen’s d* | *0.62* | *0.65* |  |  |
|  |  |  |  |  |  |
|  |  |  |  |  |  |
| HKr | Unipolar | 0.44 (0.17) | 0.44 (0.15) | *Pre-ECT: U* = 1027, *p* = 0.018  *Post-ECT: U =* 965*, p =* 0.072  *Unipolar: Z =* 1388, *p =* 0.84  *Bipolar: Z =* 86*, p =* 0.31 |  |
|  | Bipolar | 0.54 (0.19) | 0.50 (0.17) |  |  |
|  | *Cohen’s d* | *0.55* | *0.37* |  |  |
|  |  |  |  |  |  |

Data are presented as mean (SD) nmol/L.

^#^ adjusted for age, sex, BMI, smoking, presence of diabetes, presence of cardiovascular disease, use of NSAIDs, and presence of psychosis, baseline depression severity.

PAr = PA:(PL + PLP), indicative of altered vitamin B6 homeostasis towards increased B6 catabolism. HK:XA and HK:XAA are indicative of increased HK in blood owing to reduction in the activity of the B6-dependent enzymes KAT and KYNU, respectively. HKr = HK: (KYNA + XA + HAA + AA).

Abbreviations: AA, anthranilic acid; BMI, body-mass index; HAA, 3-hydroxyanthranilinic acid; HK, 3-hydroxykynurenine; KAT, kynurenine aminotransferase; KYNA, kynurenic acid; KYNU, kynureninase; NSAID, non-steroidal anti-inflammatory drug; PA, pyridoxic acid; PL, pyridoxal; PLP, pyridoxal 5′-phosphate; XA, xanthurenic acid.
