# Supplementary material for: Blood plasma B vitamins in depression and the therapeutic response to electroconvulsive therapy
Source: Brain Behav Immun Health. 2020 Mar 28;4:100063. doi: 10.1016/j.bbih.2020.100063 (PMC8474603; doi:10.1016/j.bbih.2020.100063)
Supplement: Multimedia component 4 [file mmc4.docx]

| **Supplemental Table 4**  B vitamin plasma concentrations in patients with psychotic or non-psychotic depression pre- and post-ECT | | | | | | | | |
| --- | --- | --- | --- | --- | --- | --- | --- | --- |
|  | | **Psychotic Symptoms** | **Pre-ECT** | | **Post-ECT** | **Unadjusted Statistics** | **Adjusted Statistics^#^** | |
| *B vitamins* | |  |  | |  |  |  | |
| Thiamine (B1) | | Yes | 3.65 (1.63) | | 4.71 (3.11) | *Pre-ECT: U* = 551, *p* = 0.05  *Post-ECT: U =* 700*, p =* 0.55  *Psychotic: Z =* 166, *p =* 0.08  *Non-psychotic: Z =* 1392.50, *p =* 0.82 |  | |
|  | | No | 6.41 (9.11) | | 6.40 (9.06) |  |  | |
|  | | *Cohen’s d* | *0.42* | | *0.25* |  |  | |
|  | |  |  | |  |  |  | |
| Thiamine Monophosphate (B1) | | Yes  No  *Cohen’s d* | 6.01 (2.43)  7.27 (2.85)  *0.48* | | 7.67 (3.13)  7.67 (3.13)  *0.00* | *Pre-ECT: U* = 542.50, *p* = 0.04  *Post-ECT: U =* 767, *p =* 0.10  *Psychotic: Z =* 184, *p =* 0.02  *Non-psychotic: Z =* 1582.50, *p =* 0.20 |  | |
|  | |  |  |  |  |  |  | |
|  | |  |  |  |  |  |  | |
|  | |  |  | |  |  |  | |
| Riboflavin (B2) | | Yes | 22.20 (32.71) | | 19.06 (27.55) | *Time: F*_1,92_ = 0.215, *p* = 0.64  *Group: F*_1,92_ = 0.048, *p* = 0.83  *Group×Time: F*_1,92_ = 1.24, *p* = 0.27 | *Time: F*_1,81_ = 1.55, *p* = 0.22  *Group: F*_1,81_ = 0.19, *p* = 0.66  *Group×Time: F*_1,81_ = 1.71, *p* = 0.20 | |
|  | | No | 15.97 (10.33) | | 20.18 (27.74) |  |  |  |
|  | | *Cohen’s d* | *0.26* | | *0.04* |  |  |  |
|  | |  |  | |  |  |  | |
| Flavin Monophosphate (B2) | | Yes | 12.97 (21.19) | | 12.87 (23.87) | *Pre-ECT: U* = 783.50, *p* = 0.88  *Post-ECT: U =* 741*, p =* 0.82  *Psychotic: Z =* 97, *p =* 0.77  *Non-psychotic: Z =* 1192, *p =* 0.38 |  | |
|  | | No | 9.14 (4.16) | | 8.64 (3.65) |  |  | |
|  | | *Cohen’s d* | *0.25* | | *0.25* |  |  | |
| Nicotinamide (B3) | | Yes | 961.69 (331.18) | | 963.70 (362.10) | *Time: F*_1,92_ = 0.037, *p* = 0.85  *Group: F*_1,92_ = 0.011, *p* = 0.92  *Group×Time: F*_1,92_ = 0.158, *p* = 0.69 | *Time: F*_1,81_ = 0.40, *p* = 0.53  *Group: F*_1,81_ = 0.001, *p* = 0.98  *Group×Time: F*_1,81_ = 0.26, *p* = 0.61 | |
|  | | No | 970.57 (363.98) | | 992.29 (395.06) |  |  |  |
|  | | *Cohen’s d* | *0.03* | | *0.08* |  |  |  |
|  | |  |  | |  |  |  | |
| N1-methylnicotinamide (B3) | | Yes | 106.75 (65.56) | | 126.08 (106.87) | *Time: F*_1,92_ = 0.44, *p* = 0.51  *Group: F*_1,92_ = 0.035, *p* = 0.85  *Group×Time: F*_1,92_ = 2.58, *p* = 0.11 | *Time: F*_1,81_ = 0.00006, *p* = 0.99  *Group: F*_1,81_ = 0.001, *p* = 0.98  *Group×Time: F*_1,81_ = 2.29, *p* = 0.13 | |
|  | | No | 117.45 (63.31) | | 106.97 (54.10) |  |  |  |
|  | | *Cohen’s d* | *0.17* | | *0.23* |  |  |  |
|  | |  |  | |  |  |  | |
| Pyridoxal 5ʹ-phosphate (B6) | | Yes | 43.20 (29.93) | | 44.55 (24.33) | *Pre-ECT: U* = 650.50, *p* = 0.29  *Post-ECT: U =* 758, *p =* 0.94  *Psychotic: Z =* 134, *p =* 0.52  *Non-psychotic: Z =* 1323, *p =* 0.88 |  | |
|  | | No | 53.55 (55.22) | | 50.02 (42.57) |  |  | |
|  | | *Cohen’s d* | *0.23* | | *0.16* |  |  | |
| Pyridoxic Acid (B6) | | Yes | 24.69 (12.37) | | 28.40 (11.42) | *Pre-ECT: U* = 639.50, *p* = 0.25  *Post-ECT: U =* 832, *p =* 0.55  *Psychotic: Z =* 166, *p =* 0.08  *Non-psychotic: Z =* 1451.50, *p =* 0.58 |  | |
|  | | No | 40.65 (114.16) | | 34.08 (44.78) |  |  | |
|  | | *Cohen’s d* | *0.20* | | *0.17* |  |  | |
| Pyridoxal (B6) | | Yes | 9.66 (4.41) | | 10.88 (7.77) | *Pre-ECT: U* = 764, *p* = 0.98  *Post-ECT: U =* 779, *p =* 0.91  *Psychotic: Z =* 119, *p =* 0.90  *Non-psychotic: Z =* 1417.50, *p =* 0.56 |  | |
|  | | No | 20.91 (87.27) | | 12.18 (17.78) |  |  | |
|  | | *Cohen’s d* | *0.18* | | *0.09* |  |  | |
|  | |  |  | |  |  |  | |
| *Ratios indicative of B vitamin function* | | | | | | | | |
| PAr |  | Yes | | 0.53 (0.24) | 0.56 (0.23) | *Pre-ECT: U* = 754, *p* = 0.91  *Post-ECT: U =* 867*, p =* 0.36  *Psychotic: Z =* 158, *p =* 0.14  *Non-psychotic: Z =* 1328*, p =* 0.90 |  | |
|  |  | No | | 0.55 (0.27) | 0.55 (0.22) |  |  | |
|  |  | *Cohen’s d* | | *0.08* | *0.04* |  |  | |
|  |  |  | |  |  |  |  | |
| HK:XA |  | Yes | | 6.32 (4.76) | 4.76 (2.45) | *Pre-ECT: U* = 882, *p* = 0.29  *Post-ECT: U =* 787*, p =* 0.85  *Psychotic: Z =* 103, *p =* 0.66  *Non-psychotic: Z =* 1151*, p =* 0.27 |  | |
|  |  | No | | 4.84 (2.58) | 4.93 (3.78) |  |  | |
|  |  | *Cohen’s d* | | *0.39* | *0.05* |  |  | |
|  |  |  | |  |  |  |  | |
| HK:HAA |  | Yes  No  *Cohen’s d* | | 1.75 (1.07)  1.36 (0.57)  *0.45* | 1.41 (0.56)  1.38 (0.56)  *0.05* | *Pre-ECT: U* = 905, *p* = 0.21  *Post-ECT: U =* 788*, p =* 0.85  *Psychotic: Z =* 158, *p =* 0.14  *Non-psychotic: Z =* 1276*, p =* 0.68 |  | |
|  |  |  |  |  |  |  |  | |
|  |  |  | |  |  |  |  | |
|  |  |  | |  |  |  |  | |
| HKr |  | Yes | | 0.49 (0.18) | 0.44 (0.14) | *Pre-ECT: U* = 267, *p* = 0.38  *Post-ECT: U =* 245*, p =* 0.70  *Psychotic: Z =* 84, *p =* 0.27  *Non-psychotic: Z =* 1397*, p =* 0.80 |  | |
|  |  | No | | 0.43 (0.15) | 0.48 (0.15) |  |  | |
|  |  | *Cohen’s d* | | *0.36* | *0.28* |  |  | |
|  |  |  | |  |  |  |  | |
|  | | | | | | | |  |

Data are presented as mean (SD) nmol/L. Psychotic group: *n* = 21; Non-psychotic group: *n* = 73.

^#^ adjusted for age, sex, BMI, smoking, presence of diabetes, presence of cardiovascular disease, use of NSAIDs, and depression polarity, baseline depression severity where appropriate.

PAr = PA:(PL + PLP), indicative of altered vitamin B6 homeostasis towards increased B6 catabolism. HK:XA and HK:XAA are indicative of increased HK in blood owing to reduction in the activity of the B6-dependent enzymes KAT and KYNU, respectively. HKr = HK: (KYNA + XA + HAA + AA).

Abbreviations: AA, anthranilic acid; BMI, body-mass index; HAA, 3-hydroxyanthranilinic acid; HK, 3-hydroxykynurenine; KAT, kynurenine aminotransferase; KYNA, kynurenic acid; KYNU, kynureninase; NSAID, non-steroidal anti-inflammatory drug; PA, pyridoxic acid; PL, pyridoxal; PLP, pyridoxal 5′-phosphate; XA, xanthurenic acid.
