# Supplementary material for: Blood plasma B vitamins in depression and the therapeutic response to electroconvulsive therapy
Source: Brain Behav Immun Health. 2020 Mar 28;4:100063. doi: 10.1016/j.bbih.2020.100063 (PMC8474603; doi:10.1016/j.bbih.2020.100063)
Supplement: Multimedia component 5 [file mmc5.docx]

| **Supplemental Table 5**  B vitamin plasma concentrations in remitters and non-remitters pre- and post-ECT | | | | | |
| --- | --- | --- | --- | --- | --- |
|  | **Remitter** | **Pre-ECT** | **Post-ECT** | **Unadjusted Statistics** | **Adjusted Statistics^#^** |
| *B vitamins* |  |  |  |  |  |
| Thiamine (B1) | Yes | 4.44 (2.72) | 4.90 (2.50) | *Pre-ECT: U* = 1231, *p =* 0.32  *Post-ECT: U* = 986.50, *p* = 0.39  *Remitter: Z* = 737.50, *p* = 0.33  *Non-remitter: Z* = 522.50, *p* = 0.75 |  |
|  | No | 7.33 (11.40) | 7.31 (11.52) |  |  |
|  | *Cohen’s d* | *0.35* | *0.29* |  |  |
|  |  |  |  |  |  |
| Thiamine Monophosphate (B1) | Yes | 6.76 (2.88) | 7.35 (2.52) | *Pre-ECT: U* = 1250.50, *p* = 0.25  *Post-ECT: U* = 1137.50, *p* = 0.78  *Remitter: Z =* 799, *p* = 0.12  *Non-remitter: Z* = 632.50, *p* = 0.11 |  |
|  | No | 7.24 (2.72) | 8.05 (3.67) |  |  |
|  | *Cohen’s d* | *0.17* | *0.22* |  |  |
|  |  |  |  |  |  |
| Riboflavin (B2) | Yes | 18.84 (21.90) | 18.73 (19.13) | *Pre-ECT: U* = 957, *p* = 0.28  *Post-ECT: U =* 951.50, *p* = 0.26  *Remitter: Z =* 623.50, *p =* 0.91  *Non-remitter: Z =* 422, *p =* 0.54 |  |
|  | No | 15.69 (11.79) | 21.30 (34.95) |  |  |
|  | *Cohen’s d* | *0.18* | *0.09* |  |  |
|  |  |  |  |  |  |
| Flavin Monophosphate (B2) | Yes | 11.25 (14.04) | 10.89 (15.67) | *Pre-ECT: U* = 928, *p* = 0.19  *Post-ECT: U =* 961.50, *p* = 0.29  *Remitter: Z =* 585.50, *p* = 0.62  *Non-remitter: Z =* 401, *p =* 0.39 |  |
|  | No | 8.56 (3.85) | 8.10 (3.18) |  |  |
|  | *Cohen’s d* | *0.26* | *0.25* |  |  |
|  |  |  |  |  |  |
| Nicotinamide (B3) | Yes | 948.71 (344.51) | 989 (352.74) | *Time: F*_1,92_ = 0.20, *p* = 0.66  *Group:* *F*_1,92_ = 0.68, *p* = 0.41  *Time×Group:* *F*_1,92_ = 0.01, *p* = 0.92 | *Time: F*_1,80_ = 0.39, *p* = 0.54  *Group:* *F*_1,80_ = 0.25, *p* = 0.62  *Time×Group:* *F*_1,80_ = 0.001, *p* = 0.97 |
|  | No | 991.18 (369.65) | 982.38 (425.23) |  |  |
|  | *Cohen’s d* | *0.12* | *0.02* |  |  |
|  |  |  |  |  |  |
| N1-methylnicotinamide (B3) | Yes | 115.83 (60.93) | 114.17 (54.11) | *Time: F*_1,92_ = 0.092, *p* = 0.76  *Group:* *F*_1,92_ = 1.10, *p* = 0.30  *Time×Group:* *F*_1,92_ = 0.35, *p* = 0.55 | *Time: F*_1,80_ = 0.0001, *p* = 0.99  *Group:* *F*_1,80_ = 1.57, *p* = 0.21  *Time×Group:* *F*_1,80_ = 0.002, *p* = 0.97 |
|  | No | 114.18 (67.25) | 107.92 (83.62) |  |  |
|  | *Cohen’s d* | *0.03* | *0.09* |  |  |
|  |  |  |  |  |  |
| Pyridoxal 5ʹ-phosphate (B6) | Yes | 45.28 (27.98) | 44.10 (22.77) | *Pre-ECT: U* = 1158.50, *p* = 0.66  *Post-ECT: U* = 1078, *p* = 0.87  *Remitter: Z =* 693, *p* = 0.59  *Non-remitter: Z =* 465, *p* = 0.73 |  |
|  | No | 58.01 (67.69) | 54.14 (51.72) |  |  |
|  | *Cohen’s d* | *0.25* | *0.25* |  |  |
|  |  |  |  |  |  |
|  |  |  |  |  |  |
| Pyridoxic Acid (B6) | Yes | 28.65 (10.85) | 29.86 (10.83) | *Pre-ECT: U* = 791.50, *p* = 0.02  *Post-ECT: U* = 725.50, *p* = 0.40  *Remitter: Z =* 737.50, *p* = 0.33  *Non-remitter: Z =* 578, *p* = 0.33 |  |
|  | No | 46.67 (147.23) | 36.17 (57.23) |  |  |
|  | *Cohen’s d* | *0.17* | *0.15* |  |  |
|  |  |  |  |  |  |
| Pyridoxal (B6) | Yes | 9.56 (3.40) | 9.58 (3.67) | *Pre-ECT: U* = 1009.50, *p* = 0.49  *Post-ECT: U =* 1052.50, *p* = 0.72  *Remitter: Z =* 653.50, *p* = 0.88  *Non-remitter: Z* = 516.50, *p* = 0.60 |  |
|  | No | 28.44 (112.26) | 14.51 (23.01) |  |  |
|  | *Cohen’s d* | *0.24* | *0.30* |  |  |
|  |  |  |  |  |  |
| *Ratios indicative of B vitamin function* | | | | | |
| PAr | Yes | 0.59 (0.26) | 0.61 (0.23) | *Pre-ECT: U* = 777, *p* = 0.001  *Post-ECT: U =* 750, *p* = 0.008  *Remitter: Z =* 583, *p* = 0.60  *Non-remitter: Z* = 469, *p* = 0.76 |  |
|  | No | 0.49 (0.27) | 0.50 (0.20) |  |  |
|  | *Cohen’s d* | *0.38* | *0.51* |  |  |
|  |  |  |  |  |  |
|  |  |  |  |  |  |
| HK:XA | Yes | 4.98 (3.06) | 4.94 (3.66) | *Pre-ECT: U* = 1174, *p* = 0.58  *Post-ECT: U =* 1102, *p* = 0.99  *Remitter: Z =* 646, *p* = 0.94  *Non-remitter: Z* = 624, *p* = 0.132 |  |
|  | No | 5.38 (3.44) | 4.83 (3.39) |  |  |
|  | *Cohen’s d* | *0.12* | *0.03* |  |  |
|  |  |  |  |  |  |
|  |  |  |  |  |  |
| HK:HAA | Yes | 1.42 (0.62) | 1.37 (0.52) | *Pre-ECT: U* = 1093, *p* = 0.96  *Post-ECT: U =* 1150, *p* = 0.71  *Remitter: Z =* 678, *p* = 0.70  *Non-remitter: Z* = 511, *p* = 0.85 |  |
|  | No | 1.48 (0.83) | 1.41 (0.61) |  |  |
|  | *Cohen’s d* | *0.08* | *0.07* |  |  |
|  |  |  |  |  |  |
| HKr | Yes | 0.45 (0.16) | 0.45 (0.14) | *Pre-ECT: U* = 1192, *p* = 0.49  *Post-ECT: U =* 1127, *p* = 0.84  *Remitter: Z =* 704, *p* = 0.52  *Non-remitter: Z* = 85.69, *p* = 0.27 |  |
|  | No | 0.48 (0.20) | 0.46 (0.17) |  |  |
|  | *Cohen’s d* | *0.17* | *0.06* |  |  |
|  |  |  |  |  |  |

Data are presented as mean (SD) nmol/L. Remitters: *n* = 50; Non-remitters: *n* = 44.

# adjusted for age, sex, BMI, smoking, presence of diabetes, presence of cardiovascular disease, use of NSAIDs, and depression polarity, presence of psychosis, baseline depression severity.

PAr = PA:(PL+ PLP), indicative of altered vitamin B6 homeostasis towards increased B6 catabolism. HK:XA and HK:XAA are indicative of increased HK in blood owing to reduction in the activity of the B6-dependent enzymes KAT and KYNU, respectively. HKr = HK: (KYNA + XA + HAA + AA).

Abbreviations: AA, anthranilic acid; BMI, body-mass index; HAA, 3-hydroxyanthranilinic acid; HK, 3-hydroxykynurenine; KAT, kynurenine aminotransferase; KYNA, kynurenic acid; KYNU, kynureninase; NSAID, non-steroidal anti-inflammatory drug; PA, pyridoxic acid; PL, pyridoxal; PLP, pyridoxal 5′-phosphate; XA, xanthurenic acid.
